# The potential use of bacteria and bacterial derivatives as drug delivery systems for viral infection

**DOI:** 10.1186/s12985-023-02183-z

**Published:** 2023-10-03

**Authors:** Amirhosein Faghihkhorasani, Hanan Hassan Ahmed, Noor Muhammad Mashool, Mariem Alwan, Marjan Assefi, Aya Hussein Adab, Saman Yasamineh, Omid Gholizadeh, Moein Baghani

**Affiliations:** 1 Medical Student, Iran University of Medical Sciences, Tehran, Iran; 2 Department of Pharmacy, Al-Noor University College, Nineveh, Iraq; 3 College of Nursing, National University of Science and Technology, Dhi Qar, Iraq; 4 Pharmacy College, Al-Farahidi University, Baghdad, Iraq; 5 University of North Carolina at Greensboro, Greensboro, USA; 6 Department of Pharmacy, Al-Zahrawi University College, Karbala, Iraq; 7 Young Researchers and Elite Club, Tabriz Branch, Islamic Azad University, Tabriz, Iran; 8 Research Center for Clinical Virology, Tehran University of Medical Sciences, Tehran, Iran; 9 Skin Research Center, Shahid Beheshti University of Medical Sciences, Tehran, Iran

**Keywords:** Drug delivery system, Vaccine, Bacteria, Bacterial derivatives, Viral infection

## Abstract

Viral infections in humans are responsible for fatalities worldwide and contribute to the incidence of various human ailments. Controllable targeted medicine delivery against many illnesses, including viral infection, may be significantly aided by using bacteria and bacteria-derived products. They may accumulate in diseased tissues despite physical obstacles, where they can launch antiviral immunity. The ability to genetically and chemically modify them means that vaccinations against viral infections may be manufactured and delivered to affected tissues more safely and effectively. The objective of this study is to provide an overview of the latest advancements in the field of utilizing bacteria and bacterial derivatives as carriers for administering medication to treat viral diseases such as SARS-CoV-2, hepatitis B virus, hepatitis C virus, human immunodeficiency virus, human papillomavirus, influenza, and Ebola virus.

**Graphical Abstract:**

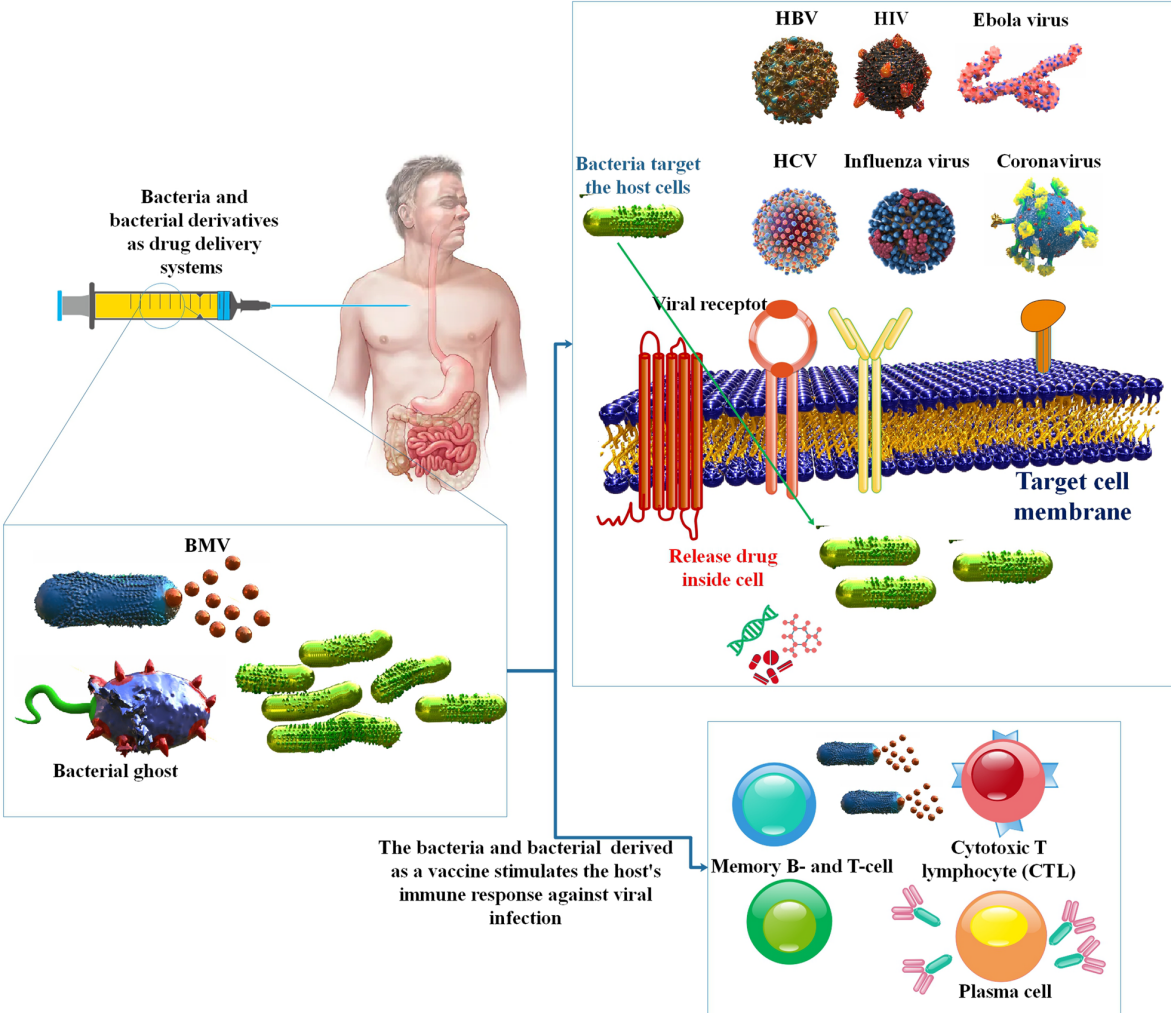

## Introduction

Epidemiological research shows that new viral infections have become more common globally in recent decades. The current pandemic outbreak of the new coronavirus is a vivid illustration of the devastating effects on the health and economic development of millions of people throughout the globe [1–3]. Measles, rubella, chickenpox/shingles, SARS-CoV, MERS-CoV, SARS-CoV-2, human immunodeficiency virus)HIV(, chikungunya virus, Ebola virus (EV), hepatitis, influenza, herpes simplex, and Zika virus all showed up in the twenty-first century without any effective antiviral treatments [4–9]. To combat these problems, nanotechnology-based drug delivery is on the rise as a means of boosting therapeutic efficacy via modifying the physicochemical features of antiviral medications [10]. The antiviral characteristics of nanoparticles (NPs) stem from their size, which speeds up drug delivery, their vast surface area to volume ratio, which allows them to carry high doses of medicines, and their malleable surface charge, which allows encapsulation [11, 12]. The practical and targeted delivery of medications, genes, and therapeutic molecules to specific organs or cells, imaging, and early-stage viral identification have all been made possible because of advances in nano-biomedical understanding. Additionally, NPs play a pivotal role in antiviral treatment by increasing the transfer of hydrophobic medicines and improving the efficiency of pharmaceutical use. Additionally, NPs play a crucial role in antiviral therapy by boosting the transfer of hydrophobic drugs and improving the efficiency of medicine use [13, 14]. For example, Clinics have utilized vaccines against SARS-CoV-2, such as lipid-based NP mRNA, inactivated virus, and recombined protein, to protect patients from contracting the virus [1, 3, 15]. In addition, another method of using synthetic biology for medicinal applications is to instruct particular bacteria to carry out certain activities. These alterations render it suitable for use as a vector for transfecting nucleic acids into the host cells as well as the delivery of medicinal compounds or proteins [16, 17]. A new biomimetic platform that can preserve natural biological functionalities is a bacterial-based medication delivery system, thanks to extensive research into bacteria and the ecosystems to which they are connected inside the human body [18–20]. Due to their unique properties, many bacterial strains have been discovered as possible medication delivery vehicles. Gram-positive pathogens like *Listeria monocytogenes* (*L. monocytogenes*) and *Clostridium novyi-NT*, as well as Gram-negative species like *Salmonella Typhimurium* (*S. Typhimurium*) and *Escherichia coli* (*E. Coli*), are included in this group [21]. A recent study suggests that both Gram-positive and Gram-negative bacteria have the potential to produce bacterial extracellular vesicles (BEVs). The two procedures generate distinct EVs, encompassing cytoplasmic membrane vesicles (CMVs), outer membrane vesicles (OMVs), outer-inner membrane vesicles (OIMVs), and outer-membrane vesicles (EOMVs). BEVs exhibit numerous advantages, including streamlined industrialization, exceptional drug delivery efficiency, facile modifiability, and straightforward bacterial infection detection. BEVs have been utilized as novel drug delivery platforms [22]. Recently, bacterial ghosts (BGs) have garnered heightened interest as promising contenders for precisely transporting biomolecules [23].

Thanks to developments in genetic engineering and synthetic bioengineering, several bacteria engineered to have minimal toxicity and great effectiveness in drug delivery have been utilized to effectively cure illnesses, particularly in viral infection treatment in animal models or clinical trials [24, 25]. So, bacteria that have been genetically modified to contain nucleic acids or genetic circuits that encode and control medicinal payloads may function as effective drug delivery systems. In-depth research is done on the procedures and objectives of strain genetic alteration. Since then, proteins, medications, enzymes, and genes have been delivered by genetically altered bacteria to treat viral infections [26]. Research has documented the efficacious production of antiviral vaccines utilizing *E. coli* for expression. This includes the expression of virus-like particle (VLP) structures of human papillomavirus (HPV) and hepatitis E virus (HEV), as well as the utilization of OMVs to transport viral antigens of dengue virus, influenza virus, and Middle East respiratory syndrome (MERS) coronavirus [27]. Experiments in which the highly conserved fusion protein (FP) of SARS-CoV-2 and porcine epidemic diarrhea virus (PEDV) were expressed on the surface of E. coli to create lethal whole-cell bacterial vaccines showed the platform's usefulness. Since a SARS-CoV-2 FP vaccine afforded cross-protection against PEDV, the FP may be an attractive target for a broadly protective coronavirus vaccine. This low-cost vaccination platform has the potential for usage in underdeveloped nations when combined with a vaccine-appropriate bacterial vector [28]. Additionally, because of their bio-adhesive qualities, BGs may attach to host cells and transfer their encapsulated payload of medications or biomolecules to the desired location. Female BALB/c mice were used to study the delivery of hepatitis B virus (HBV) core 149 (HBcAg-149) proteins attached to the IM or OM of *E. coli*. Evidence demonstrated that bacterial apparitions successfully transmitted HBcAg-149 to the experimental animals. Because of the innate immunogenicity of the Gram-negative bacterial cell membrane, BGs also provide a viable route for plasmid DNA immobilizations, making them a unique carrier [29].

In the present study, we explore the potential of diverse bacteria and bacterial derivatives as vehicles for drug delivery in various viral infections, including but not limited to SARS-CoV-2, HBV, HCV, HIV, HPV, influenza, and EV.

## Bacterial strains as drug delivery systems

As genetic engineering, synthetic biology, and knowledge of host–pathogen interactions have advanced rapidly in recent years, bacteria have become valuable tools for treating various disorders. The vast majority of bacteria found within the human body are harmless, and certain strains can be engineered to become “smart” living therapeutics with specific properties for treating various diseases [30]. To function as a viable in vivo intracellular delivery approach, an ideal bacteria-based delivery system must be capable of performing three essentials’ functions. Firstly, the bacteria must be able to target a disease-relevant cell type, such as epithelial cells. Secondly, they must be able to enter the target cell upon arrival. Finally, they must be capable of escaping the phagosome to deposit their cargo into the cytoplasm. Neutrophils, classified as professional phagocytic cells, can engulf bacteria via phagocytosis effectively [30, 31]. However, certain cell types, including epithelial cells, potential targets for therapeutic intervention, do not possess inherent phagocytic capabilities. Consequently, it is necessary to modify the bacteria to enhance the process of cellular uptake [32]. In recent years, scientists have genetically altered bacterial species to make them safer and more effective in treating a wide range of ailments. The specificity of bacterial-based treatment techniques relies heavily on the particular qualities of these bacteria, such as their ability to localize to the location of a disease and their ability to produce therapeutic chemicals. The bacterial genera examined in preclinical and clinical treatment trials encompass *Streptococcus*, *Clostridium*, *Bifidobacterium*, *Listeria*, *Escherichia*, *Lactobacillus*, and *Salmonella* [33]. For treating gastrointestinal disorders, for instance, a synthetic microbe may be orally administered, travel to the affected area, engrafted, and begin medication delivery. In the case of protein-based substances, this is particularly helpful since they would usually be destroyed by the stomach’s acidic environment. As a combination treatment, many therapeutic chemicals may be generated by a single cell simultaneously. Producing and delivering the active compound(s) in vivo through a synthetic chassis reduces treatment costs by eliminating the requirement for purification and formulation [34]. In addition, developingsynthetic bacterial “physicians” requires a thorough understanding of both the ecology of healthy bacterial colonies and the genetic underpinnings of human disorders [35]. Furthermore, treatments that are less expensive than conventional pure antigen formulations might be substituted for synthetic bacterial antigen delivery systems. A protective antigen from *Bacillus anthracis* linked to a peptide that targets dendritic cells is expressed by *Lactobacillus acidophilus* thanks to genetic engineering. The synthesized strain offered the same infection protection as pure antigen after being given as an oral vaccination. Additionally, viral infections may be avoided by using artificial microbes. When evaluated in a mouse model, an *S. Typhimurium* DNA vaccine delivery system for influenza immunization produced complete immunity. By introducing various hemagglutinin and neuraminidase combinations, this vaccination method has the additional benefit of to respond to newly developing influenza strains quickly. The vaginal commensal *Lactobacillus jensenii*, modified to produce the HIV-1 entrance inhibitor protein cyanovirin-N, serves as a second example of defense against pathogenic viruses. Repeated simian HIV challenges to macaques colonized with this strain resulted in a 63% decrease in the acquisition and a sixfold reduced viral load in macaques with breakthrough infection [35].

## Bacterial-derived-based drug delivery methods

Recent advancements in biotechnology include medicine delivery systems as well as nucleic acid and other biomolecule delivery systems. Designing and creating innovative targeted delivery methods is gaining popularity. The variety of bacteria employed as therapeutic and vaccine-delivery vehicles has increased with the development of genetic tools and expression systems. The modalities of distribution used by bacteria-derived medicines and vaccines include secretion, membrane vesicles (MVs) or BGs, surface display, and cell lysis; each has benefits and problems of its own [36]. Bacteria have been used for decades as medicinal and vaccine delivery systems. But traditionally, there have only been a few strains of bacteria utilized. The development of genetic tools and advances in immunology have increased our ability to design bacteria using various delivery methods. Each distribution technique has particular considerations, optimization, and safety issues depending on the application [36]. Ghosts of *E. coli* bacteria, which represent vacant bacterial cell envelopes, have been studied, specifically their two membrane compartments. Mice were immunized subcutaneously to test the efficacy of BGs anchored in the inner or outer membrane of E. coli that contained the HBV core 149 protein (HBcAg-149) as a model antigen. Immune responses against the HBcAg-149 target antigen were greatly enhanced by both methods in mice. The findings suggest that BGs are a promising antigen-delivery mechanism [37]. In another work, scientists used modified vesicle technology to develop synthetic bacterial vesicles (SyBV) to activate the immune system without the significant immunotoxicity of OMV. SyBV was created from bacterial membranes employing detergent and ionic stress. Compared to natural OMV, SyBV elicited fewer inflammatory responses in macrophages and mice. Immunization with SyBV or OMV produced comparable levels of antigen-specific adaptive immunity. Mainly, immunization with SyBV from *Pseudomonas aeruginosa* shielded mice against bacterial challenge, followed by a significant decrease in lung cell infiltration and inflammatory cytokines. Additionally, immunization with SyBV generated from *E. coli* shielded mice against *E. coli* sepsis similarly to the OMV-immunized group. The induction of B- and T-cell immunity contributed to SyBV’s protective effect. Additionally, SyBV were modified to exhibit the SARS-CoV-2 S1 protein on their surface, and these vesicles elicited responses from T-cells and antibodies specific to the S1 protein. These findings show that SyBV may be a reliable and effective vaccination platform for avoiding bacterial and viral diseases [38]. Another work showed that two distinct SARS-CoV-2 antigens could be conducted concurrently in the lumen and on the surface of modified OMVs utilizing a bivalent antigen display platform built on mCherry and GFP. Comparing immunogenicity, OMVs delivering either the ClyA-NG06 fusion or RBD alone induced a weaker humoral response in mice than those providing ClyA-NG06 fusion and RBD. Researchers demonstrated a practical method for displaying SARS-CoV-2 antigens in the lumen and on the surface of the same OMV, and they emphasized the potential of OMVs as generic multi-antigen carriers [39]. A variety of characteristics of bacterial MVs (BMVs) make them attractive candidates for vaccine development. This includes their capacity for displaying proteins from many sources, PAMPs in their natural state for inducing potent immune responses, their nanoscale size for effective antigen delivery and processing, and their adaptability to be further modified as required. Although BMV nano vaccines have shown a lot of promise, there are still a lot of obstacles in the way of their clinical use. First off, as they have to be extracted from live bacteria, there is a built-in batch-to-batch variability in size, antigen density, and general composition that needs to be taken into consideration during large-scale manufacture. The standardization of BMV collection methods and the use of bacterial stocks with low passage numbers are methods to reduce variation. It is necessary to research to provide improved isolation and purification methods that can accurately distinguish dangerous bacterial components from BMVs [40].

### Bacteria secretion

The majority of medicines are delivered through the bacteria’s built-in secretion system. Secretion-based drug delivery helps keep bacteria healthy during host interactions. Diffusion of effector molecules via tight junction gaps has been postulated to contribute to systemic delivery. Close physical relationships between the delivery vehicle and host cells at the epithelial barrier have been proposed to play a role in this process [36]. Protein secretion, in which proteins are transported from the cytoplasm to various locations within the cell, the environment, and/or other bacteria or eukaryotic cells, is a fundamental activity of prokaryotic cells. Prokaryotes have evolved various protein transport mechanisms, most of which rely on specialized protein secretion systems. Bacterial growth relies on protein secretion systems, which have many different functions. Some secretion systems are widespread and secrete many different substrates in different bacterial species, whereas others are rare or exclusive to the secretion of just a few proteins. Some bacterial infections utilize specialized secretion systems to influence the host and carve out a suitable environment to reproduce [41]. Furthermore, it was shown that systemic IL-22 levels were elevated when mice were orally administered recombinant probiotics secreting IL-22. Recent clinical studies for bacterial secretion of effector molecules have included an unsuccessful Phase 2 study (NCT03234465ii) testing the effectiveness of *Lactococcus lactis* (*L. lactis)* (AG013), which secretes human Trefoil Factor 1, in treating oral mucositis. In Phase 2a clinical study, *L. lactis* (AG019), which secretes hPINS and hIL-10, is being investigated for treating type I diabetes (NCT03751007iii, 32). In another study, an *L. monocytogenes* strain secreting an antigen-adjuvant f FP (tLLO-HPV-16 E7) is now undergoing a Phase 3 clinical study to treat cervical cancer (NCT02853604i). In contrast, live vaccine administration through secretion has advanced considerably as a method of delivery [36, 42].

Bacterial ATP-binding cassette (ABC) transporters can secrete numerous heterologous proteins, given that they are fused with the appropriate C-terminal signal sequence. However, specific proteins cannot be secreted despite possessing the correct signal sequence. The invention of a method to secrete these non-secret able proteins would be valuable both for understanding the secretory physiology of ABC transporters and for industrial applications. Researchers hypothesize that ABC transporters cannot secrete their target substrate protein due to the presence of cationic “supercharged” areas within the protein. The bacteria *Pseudomonas* fluorescens is helpful for this purpose. Researchers have also suggested neutralizing the cationic supercharged regions of such substrate proteins by structure-preserving point mutational studies as a means of rescuing their secretion. Cationic supercharged areas had their cationic charge density lowered by introducing anionic or neutral hydrophilic amino acids instead of projecting non-structural cationic amino acids. Researchers have cited various rescued secretions, including the SARS-CoV-2 spike protein, glutathione-S-transferase, streptavidin, lipase, tyrosinase, cutinase, growth factors, etc. The present study represents a noteworthy contribution to our comprehension of the physiological attributes of protein secretion that rely on ABC transporters. Furthermore, it establishes a foundation for developing a protein-producing platform based on secretion by providing a means to anticipate secretability and a mechanism to restore secretion by eliminating regions that impede it [43].

### Bacterial extracellular vesicles

**Fig. 1:**
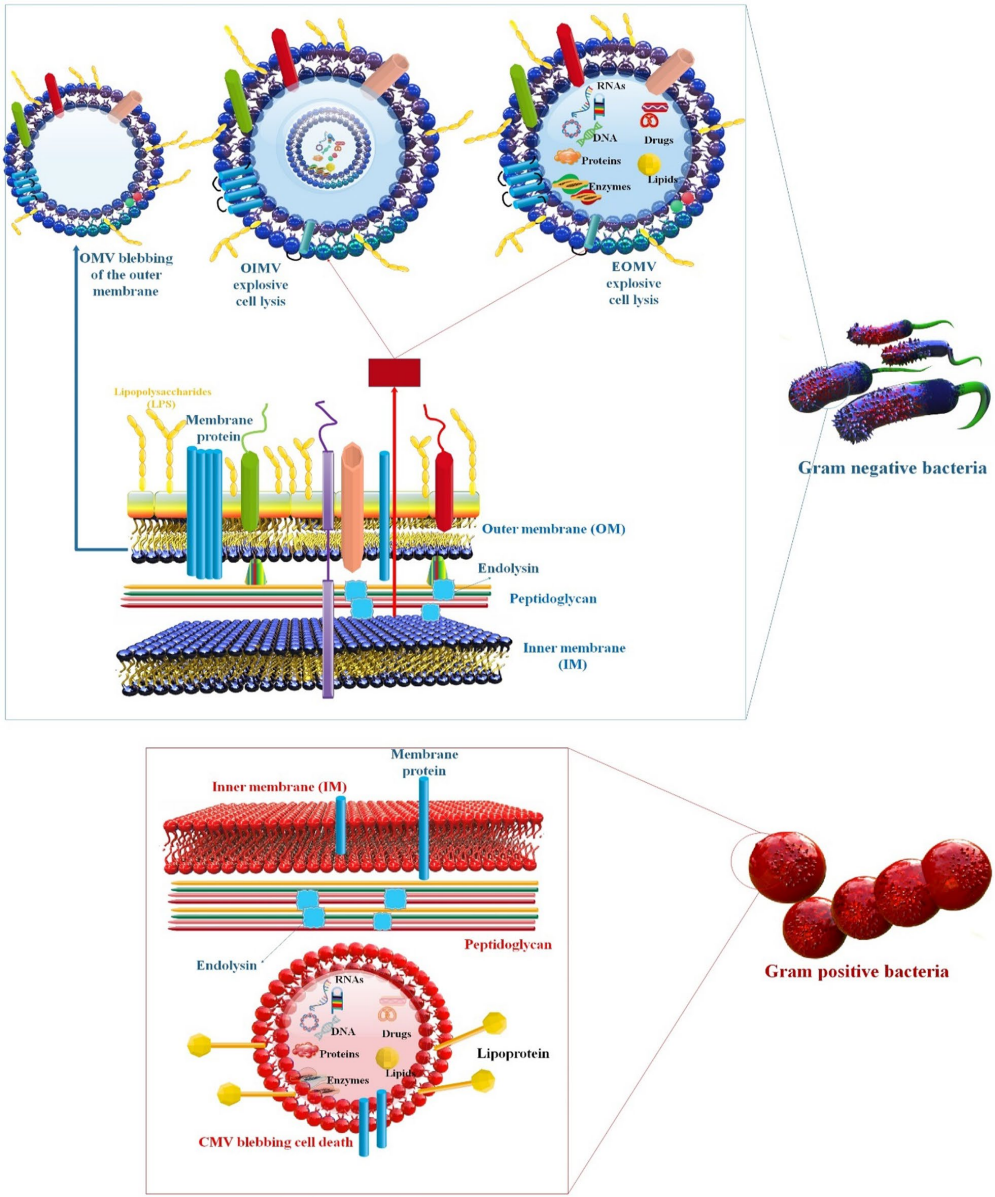
BMV biogenesis and load kinds, comparing Gram-negative and positive bacteria. Researchers offered the organization of OMVs because of the agglomeration of phospholipids due to the elimination or reduced expression of vacj and yrb genes. Novel investigation proposed a novel pathway resulting in the organization of vesicles named the outer-inner membrane vesicles (OIMVs) and explosive outer-membrane vesicles (EOMVs) based on explosive cell lysis activated through the enzymatic action of endolysins [50]. Gram-negative BMVs are produced via two major models, blebbing of the OM and explosive cell lysis. The insertion of hydrophobic molecules or the inconsistency of peptidoglycan biosynthesis into the OM causes blebbing of the OM, which generates classic OMV. The weakness of the peptidoglycan layer through endolysin leads the IM to protrude into the periplasm, which develops explosive EOMV or OIMV. In addition, the biogenesis mechanism of gram-positive BMVs release is bubbling cell death. The endolysin destroys the peptidoglycan layer and leads to bubbling cell death in gram-positive bacteria, and generates CMVs. The difference in loads of gram-positive and negative BMVs goes beyond the presence of LPS and contains other molecules, including nucleic acids, proteins, lipids, and metabolites [44]

EVs of nanoscale dimensions, which originate from bacterial sources, possess a wide array of contents and can transfer bioactive molecules between cells. The promising intercellular interactions exhibited by BEVs derived from cell membranes suggest their potential as innovative platforms for drug delivery [44]. Gram-negative and Gram-positive bacteria, like human cells, release nanoscale MVs into the extracellular environment either constitutively or in a controlled way. These BEVs are spherical, bilayered proteolipids loaded with virulence factors, bioactive proteins, lipids, and nucleic acids. Recent developments in this area confirm that these vesicles play crucial pathophysiological roles in interactions between bacteria and their hosts as well [45]. The BEV surface may now be modified and embellished with various proteins and NPs thanks to recent breakthroughs in biotechnology. BEVs are being examined as vaccines, cancer immunotherapy drugs, and CDVs and have drawn substantial attention from various biological sectors. To realize their full therapeutic potential, considerable obstacles regarding their safety, effectiveness, and mass manufacturing must be overcome [46]. The significance of bacterial OMVs has been identified in facilitating bacterial communication, sustaining bacterial adaptability and viability in the host milieu, and promoting bacterial physiology and pathogenicity. Numerous research endeavors have concentrated on vaccines based on vesicles and have utilized these characteristics to showcase the potential of OMVs as viable vaccine contenders against bacterial infections [47]. In addition, Minicells are small vesicles that lack a nucleus and have a size range of 100–400 nm. These vesicles are formed due to the deactivation of the Min operon, which regulates the typical process of bacterial cell division. As a result, the formation of minicells inhibits the formation of polar sites for cell fission. Minicells, which account for roughly 20% of the bacterial cell volume, play a significant role in removing damaged or oxidized proteins, thereby enabling bacteria to withstand higher concentrations of antimicrobial agents, such as streptomycin [48, 49] (Fig. 1).

### Bacteria lysis and bacterial ghosts

Prophages are a type of bacterial virus that remains in a latent state, with their genomes integrating into the genome of the host bacterium. Prophages are prevalent in both commensal bacteria and probiotics, and they exhibit a dynamic function in vivo bacterial fitness. For example, Bacteriophages are released from a subset of *L. reuteri* cells when their prophages are activated, as happens during GI transit. By eliminating bacteria susceptible to these bacteriophages, *L. reuteri* gains a competitive edge [51]. The production of a phage-derived lysin (lysA) improved the lysis of *L. monocytogenes* which was delivering a DNA vaccine. In contrastan *E. coli Nissle* strain designed to deliver intracellularly accumulated checkpoint inhibitors to cancer cells was lysed using a quorum-sensing-driven lysis circuit. To effectively mediate the release of medicines from bacteria, phage, and their lytic processes are thus very effective [52].

Once assumed to simply represent the buildup of sufficient lysozyme activity throughout the infection cycle, it has recently been demonstrated that the lysis of bacterial hosts by double-strand DNA bacteriophages is a highly managed and temporally planned process. There are three stages for Gram-negative phages, one for each of the three layers of the cell envelope (ENV) that are compromised during infection: the inner membrane (IM), the peptidoglycan, and the outer membrane (OM). The process is under cytoplasmic membrane-level regulation. Canonical lysis involves the accumulation of a phage-encoded protein called holing in the cytoplasmic membrane without causing any damage until it is triggered at an allele-specific moment, at which point it punctures the membrane at the micron scale. The soluble endolysin may leave the cytoplasm and begin degrading the peptidoglycan [53].

**Fig. 2:**
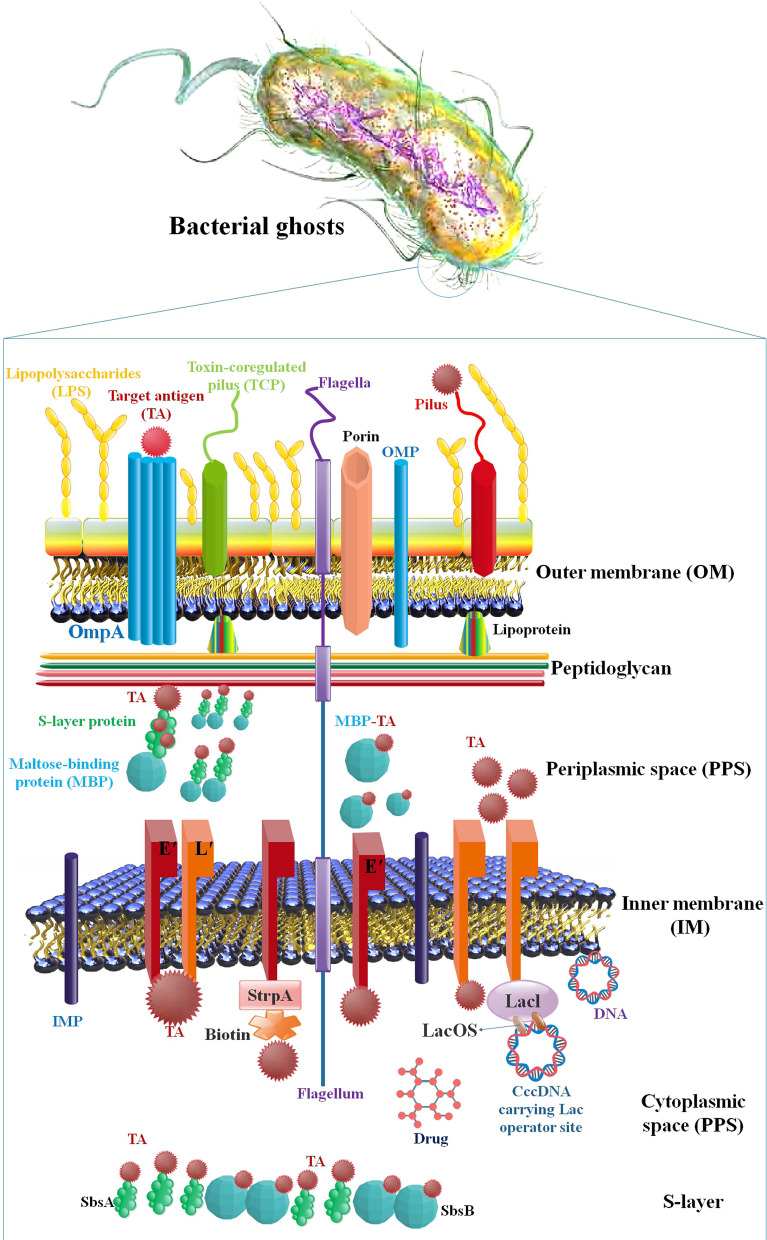
Schematic of localization of several molecules in BGs. Several techniques for antigen presentation in the BG envelope complex—BG themselves transport native antigens, such as lipopolysaccharide (LPS), outer membrane protein (OMP), inner membrane protein (IMP), toxin coregulated pilus (TCP), flagella, pili—target antigen (TA) may be presented on the cell surface through fusion with OmpA—the PPS can be encapsulated with target antigen via maltose binding protein (MBP)-SbsA- FPs, through fusion of the target antigen with MBPor as sole target antigen utilizing the gene III signal sequence. Protein target antigen may be combined into the inner membrane through E′, L′ or E′/L′-anchoring, biotinylated antigen can be bound to E′-FXa-StrpA membrane anchors, DNA carrying the lac operator site can be bound to L′-anchored lacI suppressor molecules—target antigen attached with SbsA-/SbsB proteins develop S-layers in the PPS [59]

Protein-E-mediated lysis of Gram-negative bacteria yields BGs, essentially empty non-denatured ENVs that maintain all of the normal cell’s morphological and structural properties. Because they include naturally occurring immune-stimulating chemicals such as LPS, lipid A, and peptidoglycan, they may be exploited as vaccine candidates in and of themselves [54]. One lysis gene, E, encoded by bacteriophage PhiX174, is adequate for inducing lysis of *E. coli* [55]. E-mediated lysis has been achieved in various Gram-negative bacteria, including *E. Coli K12*, enterohaemorrhagic (EHEC) and entertoxigenic (ETEC) strains, *Actinobacillus pleuropneumoniae, Bordetella bronchiseptica*, *Erwinia cypripedii*, *H. pylori*, *Klebsiella pneumoniae*, *Mannheimia haemolytica*, *Pasteurella multocida*, *Pseudomonas putida*, *Ralstonia eutropha*, *S. Typhimurium* and *enteritidis* strains, and *Vibrio cholerae* (*V. cholerae*). This diverse range of bacteria demonstrates the general applicability of E-mediated lysis to all Gram-negative bacteria, with the caveat that the E-specific lysis cassette must be delivered to the new recipient using a vector that permits precise suppression and induction control of the fatal gene *E* [56]. Pre-lysis localization of target antigens by BG is possible in the OM, the IM, the periplasmic space (PPS), and the interior lumen of the cytoplasmic space (CPS), as will be detailed in greater depth below. S-layer FPs, produced as shell-like self-assembly structures filling either the PPS or CPS, may be used to attach foreign target antigens to the OM or IM, export them into the PPS, or all three. The OM is an asymmetric lipid bilayer composed of lipopolysaccharide (LPS) on the outside and phospholipids on the inside. From the OM, polysaccharide molecules such as LPS, filaments, and pili reach out into the surrounding space [57] (Fig. 2). Success in several illness conditions and as allergen vaccines have been proven by intracellular accumulation of medicines for in situ administration. In these applications, the lysis mechanism(s) is/are unknown.* L. lactis* is an example of a noncommercial bacterium that has not developed to withstand the challenging environment of the human GI tract. Therefore, it is probable that spontaneous, stress-induced lysis and/or breakdown of the cell wall caused *L. lactis* to release intracellular therapeutic. Because *L. lactis* MG1363, a frequently used strain for biotherapeutic administration, lacks an active prophage, prophages cannot induce *L. lactis* MG1363 to lyse [58].

The capacity to colonize and target human tissues, boost the immunogenicity of vaccines, and have excellent loading capacities are just a few of the numerous benefits these delivery platforms retain from bacteria. There has been a recent resurgence of interest in BGs as a possible method of targeted biomolecule delivery [60]. The BGs refer to bacterial cells that belong to the Gram-negative category. These cells have undergone a process of controlled expression of the cloned bacteriophage PhiX174 gene *E*, which has resulted in the expulsion of their cytoplasmic contents. BGs are just as antigenic as their mother cells because they maintain the architecture and components of the normal bacterial cell wall. Due to their excellent loading capacity in their inner compartments, BGs may be used as effective delivery vehicles for subunit vaccines or DNA vaccines, medications like chemotherapeutics, and antibiotics, all while retaining the morphology of the original bacterial cell. BGs are seen as a promising technological platform for creating vaccines and therapeutic drug carriers for treating both infectious and non-infectious disorders [61]. In treating tumors, BGs are an effective advanced drug delivery system (ADDS) for the administration of hazardous chemicals. BGs provide a platform for the development of novel forms of (polyvalent) CDVs due to their internal space, which may be loaded with single components or mixtures of peptides, medicines, or DNA. CaCo2 cells effectively released Dox from endo-lysosomal compartments and accumulated it in the nucleus after taking BGs laden with Dox. After cells internalized Dox-loaded BGs, their viability and proliferative capability were drastically reduced (by two to three orders of magnitude) compared to those treated with free Dox. Leukaemia cells were subject to the same observation. In addition, melanoma cells demonstrated an impressive capacity for internalizing BGs. BGs are effective against several cancer kinds. The potential of BGs as DNA delivery vectors has also been studied. Both professional APCs and tumor cells have been shown to phagocytose and internalize DNA-loaded BGs, with up to 82% of cells expressing the plasmid-encoded reporter gene [62].

### Bacteria membrane vesicles and bacterial ghosts

**Fig. 3:**
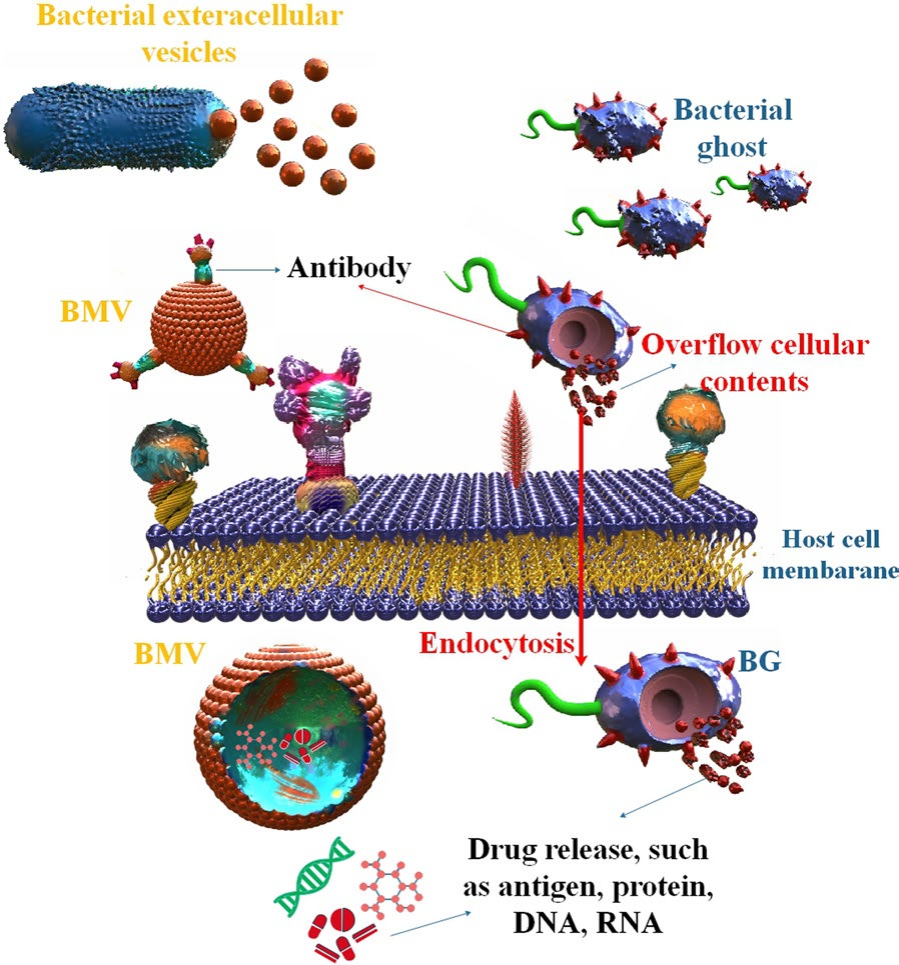
BG and BMVs as a drug delivery system

Both MVs and BGs are lipid membranes, which means they can hold tiny organic substances like DNA and RNA. The ability to emit MVs to traffic signal molecules has developed spontaneously in several bacterial species. Eukaryotic cell delivery of poisons and anti-growth factors. Some dangerous bacteria use MVs to share virulence genes and antibiotic resistance genes between themselves [63]. While MVs exist in nature, BGs need human intervention to create. BGs are empty bacterial cell ENVs produced when the cell membrane of Gram-positive or Gram-negative bacteria is broken. Stability during room temperature storage is a clear benefit of MVs/BGs as a therapeutic chassis. While MVs exist in nature, BGs need human intervention to create. BGs are empty bacterial cell ENVs produced when the cell membrane of Gram-positive or Gram-negative bacteria is broken. Stability during room temperature storage is a clear benefit of MVs/BGs as a therapeutic chassis [36, 64]. Antigens may be explicitly delivered to immune cells, and the vaccines MV/BG can show immunogenic components on their surface to do so. Due to the relative lack of regulatory requirements for MVs/BGs compared to live biotherapeutic products, such as antimicrobial sensitivity testing, drug-drug interaction detection, and virulence factor screening, it is anticipated that MV/BG-based vaccines will have fewer adverse effects on patients. Unlike live bacterial delivery systems, which also manufacture therapeutic chemicals, MVs are only carriers. Therefore, before delivery, MVs/BGs need to take extra technical procedures to encapsulate the therapeutic payload in the vesicle [36] (Fig. 3).

### Surface display

Both pathogenic and nonpathogenic bacteria have immunostimulatory properties, primarily mediated by proteins found on the surfaces of the bacteria or their spores. By using bacteria's capacity for immunomodulation, the surface display of recombinant antigens or antibodies (Abs) on bacteria may function as effective vaccinations. Vaccinations that promote both humoral and cell-mediated immunity often result in more potent immune responses than vaccinations that just promote one form of protection. By conveying antigen to activate humoral immunity, surface antigen presentation uses the bacterial surface's inherent capacity to stimulate cell-mediated immunity [65, 66]. Therefore, attenuated pathogens are often employed for this kind of bacterial treatment. Vaccines made from *L. lactis* were studied to see if antigen degradation affected their ability to provoke an immune response, and researchers found that HPV-16 E7 protein surface display elicited a more robust immune response from cytotoxic T lymphocytes than *L. lactis* either secreting or accumulating E7. While the surface display on spores and pathogenic bacteria is more generalizable, probiotic bacteria that do so show significant potential as vaccine-delivery vehicles. Phase 2 clinical trials now test *Lacticaseibacillus casei* exhibiting HPV E7 protein coupled to transmembrane protein PgsA [67].

## Bacteria and bacterial derivatives as drug delivery systems in viral infection

Novel approaches to treating viral illnesses must be developed. Drug delivery methods based on nanomaterials are attractive options in the search for therapeutic advantages because of their distinctive properties. Researchers should also examine how the most prevalent viruses, such as the flu, HIV, SARS-CoV-2, monkeypox, and HPVs, are being treated and prevented using the latest advances in nanomedicine [4, 17, 68, 69]. Bacterial entry into mammalian host cells involves various mechanisms, such as clathrin-mediated endocytosis, caveolin-mediated endocytosis, and membrane fusion. MVs, prominent spherical liposomes, play various roles in Gram-positive and Gram-negative bacteria. These roles include intercellular communication and the transportation of secreted proteins and virulence factors into the host or competing bacterial cells [70]. In addition, it is possible to utilize genetically modified bacteria to suppress particular infectious ailments [71].

The concept that the most effective approach to managing prevalent infectious diseases is by developing potent vaccines that do not require adjuvants has led to the exploration of diverse biological immune-stimulating constituents as novel vaccine contenders. In contemporary times, there has been a surge of interest in EVs, commonly referred to as exosomes and microvesicles in mammalian cells and OMVs in Gram-negative bacteria, as potential candidates for the next-generation vaccine. Nevertheless, the greater the invasiveness and efficacy of the vaccine in administration, the higher the likelihood of severe immune toxicity [72]. The immune system reacts aggressively to the live bacterial vector because of its inherently hostile characteristics. The mucosal method is used to deliver the live bacterial vector, which has the extra advantage of boosting the immune system both mucosally and systemically. The vaccination is straightforward to deliver through the mucosal route, which is less intrusive and increases patient acceptability. One of the most promising microorganisms utilized as a live bacterial vector is the lactic acid bacterium. *Salmonella spp.* and *Shigella spp.* are two examples of attenuated harmful bacteria employed as DNA vaccine carriers. Several studies have shown that live bacterial vectors are an effective method for delivering DNA vaccinations [73]. Furthermore, BG's potent tropism for APCs encourages the development of cellular and humoral responses to heterologous antigens and carrier- ENV complexes. BGs are well-suited for use as combination vaccines because of the ease with which they may be produced and packaged with (many) target antigens. Because they are freeze-dried, BG vaccines have a long shelf life and don’t require cold-chain storage; they’re risk-free because they don’t use host DNA or live organisms; they’re more effective at delivering their target antigens than traditional methods; they’re flexible in terms of the DNA or protein antigens they can use; and they're highly bioavailable [56]. Even though BG have been studied as vaccine candidates for use against their own ENV structures, they are most often utilized as flexible carriers and adjuvant vehicles for antigens of bacterial or viral origin. A proficiently crafted bacterium engineered for drug delivery purposes comprises three fundamental constituents: the localization of the affected tissue and regulatory input cue, the intracellular genetic circuitry component, and the active compound delivery module [16]. The present study aimed to examine the potential of bacterial-mediated drug delivery systems in treating viral infections, including but not limited to hepatitis virus, coronavirus, HIV, influenza, and EV.

### The utilization of bacteria as a delivery system in SARS-CoV-2

Coronaviruses (CoVs) are classified into four distinct phylogenetic groups, namely Alpha-, Beta-, Gamma-, and Delta-CoV. Additionally, human CoVs can be primarily categorized into two types, namely α and β-CoV [74–76]. SARS-CoV-2 is classified as a member of the β-coronavirus family and is responsible for the global COVID-19 pandemic [77, 78]. The virus is known to cause both upper and lower respiratory tract illnesses, including asymptomatic cases [79, 80]. CoVs are positive sense, single-stranded RNA (+SS-RNA) [81]. The viral genome contains multiple smaller open reading frames (ORFs). ORFs in question are predicted to be responsible for encoding various structural proteins, such as the spike (S) glycoprotein, ENV, membrane (M), nucleocapsid (N) proteins, as well as non-structural proteins (NSP) [82]. According to research, *Salmonella* strains have the potential to serve as a promising foundation for the creation of an oral vaccine for COVID-19. This approach could serve as a viable alternative for addressing the emergence of diverse mutated coronavirus strains and novel infectious diseases in the future. Bacteria-based vaccines can be developed through oral administration of bacterial strains that exhibit tolerance to the gastrointestinal tract. In a study, an attenuated strain of Salmonella (characterized by diminished virulence and toxicity) was investigated as a potential carrier for oral vaccines. The present strain was engineered with the aim of producing or partially releasing antigens against SARS-CoV-2 through the utilization of the sipB160 protein. Significantly, *Salmonella* has the potential to function as a vaccine by inducing activation of B- and T-cells through phagocytosis or viral infection of macrophages that are present in the intestinal region while maintaining favorable bacterial cell viability. The synthesis of three distinct antigens was conducted utilizing bioinformatics data about the genetic makeup of antigens to facilitate the development of COVID-19 vaccines. A plasmid using the lac operon was developed as a vector for expressing an antigen in *Salmonella*. Bacteria were transformed with the synthesized version of the intended plasmid. Here, the plasmid was first confirmed cloned in *E. coli* and then converted into a shuttle Salmonella strain and a vaccine-grade *Salmonella* strain. Three different coronavirus protein antigen candidates were generated to ensure the antigen was adequately expressed in the vaccination candidate strain [83].

**Fig. 4:**
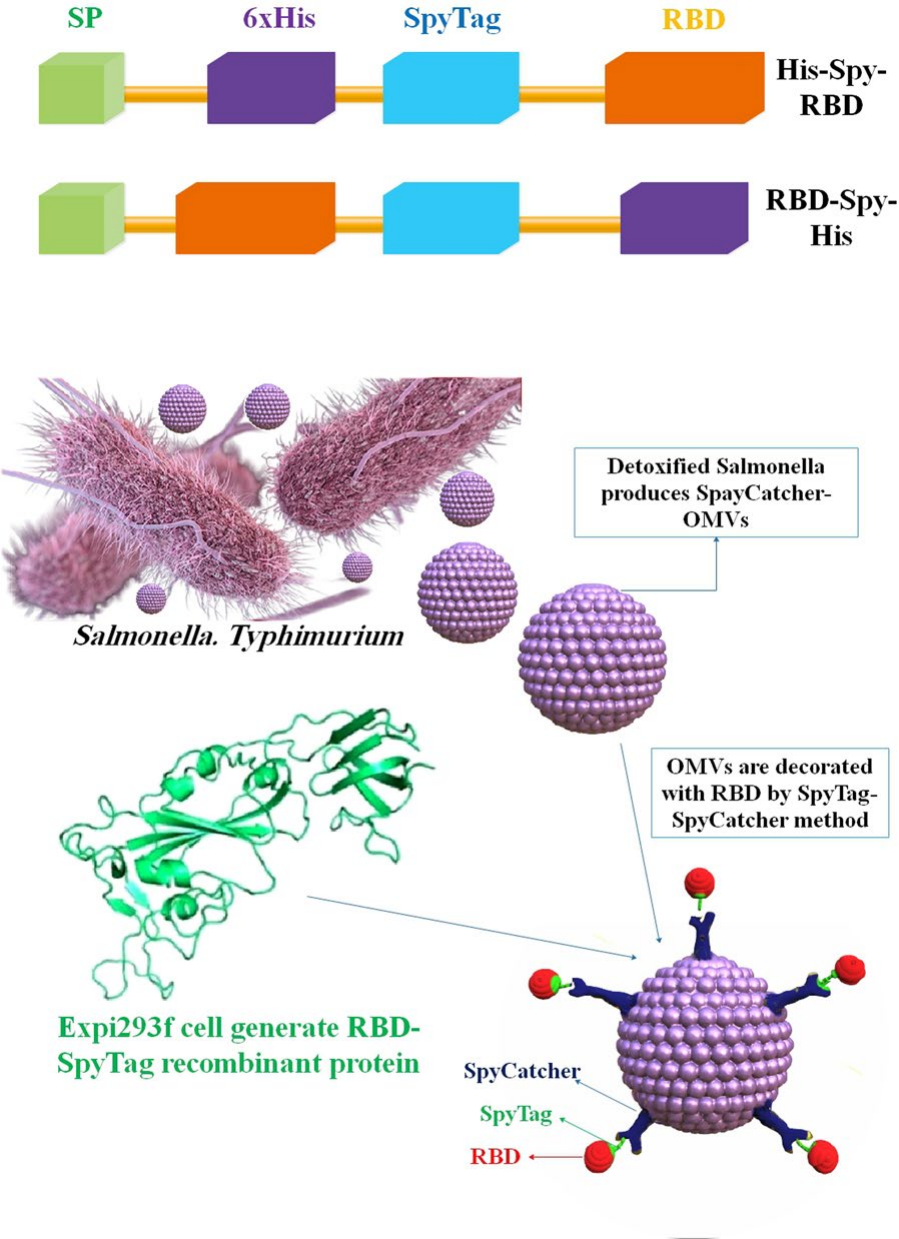
Outline of the building blocks of expressiveness and the adornment of the OMV. Recombinant antigens based on RBDs fused to the N and C termini of SpyTag are designed. A simplified diagram showing how RBD-OMVs are made

OMV-based vaccinations will likely be much simpler to store and carry than, for example, mRNA vaccines; nonetheless, stability and effectiveness must be studied rigorously for each formulation. These qualities may advocate for the widespread use of OMV vaccines, particularly in regions where low-temperature refrigeration technology is scarce. Two other OMV-based SARS-CoV-2 vaccines have been brought to researcher attention since our preprint was published [84]. In a study, a novel SARS-CoV-2 vaccine candidate has been reported by researchers, which utilizes *S. Typhimurium* EVs decorated with the Spike receptor-binding domain (RBD) derived from mammalian cell culture. The *Mesocricetus auratus*, commonly known as the golden Syrian hamster, was utilized as a COVID-19 model and subsequently administered immunization via RBD-conjugated OMVs (RBD-OMVs). The administration of intranasal (i.n.) immunization elicited elevated levels of blood anti-RBD IgG and observable mucosal responses. The vaccinated individuals exhibited neutralizing antibody activity against wild-type and Delta variants. When exposed to live virus, hamsters that were immunized with RBD-OMV showed a significant reduction in body mass loss, lower virus titers in bronchoalveolar lavage fluid, and less severe lung pathology compared to animals that were immunized with unconjugated OMVs or vehicle control. The findings of this research underscore the significance and adaptability of vaccine strategies based on OMVs [85] (Fig. 4). In another study, an i.n. COVID-19 subunit vaccine was developed by Van Der Ley et al. based on a recombinant, six-proline-stabilized, D614G spike protein of SARS-CoV-2 connected through the LPS-binding peptide sequence mCramp (mC) to OMVs from *Neisseria meningitidis* (*N. meningitidis*). After the spike protein was produced in CHO cells and coupled to the OMVs, mice and Syrian hamsters received i.n. or intramuscular (i.m.) prime-boost immunizations with the OMV-mC-Spike vaccine. The administration of OMV-mC-Spike resulted in the production of serum-neutralizing antibodies in all vaccinated animals, as observed in this study. It is noteworthy that i.n. vaccination resulted in elevated levels of spike-binding IgG and IgA Abs in the nasal and pulmonary regions, whereas i.m. vaccination only elicited an IgG response in the serum. The study findings indicate that hamsters, who were administered the second vaccination dose against SARS-CoV-2, were safeguarded against weight loss and viral replication in the lungs. This was in contrast to the control groups that received OMV or spike alone. Hamsters vaccinated against OMV-mC-Spike exhibited no pathological lesions in the lungs 7 days after the challenge, in contrast to the unvaccinated controls. Positive results from clinical trials with the OMV-mC-Spike candidate vaccine indicate that this unique, needle-free, subunit vaccination idea should be developed further [86].

**Fig. 5:**
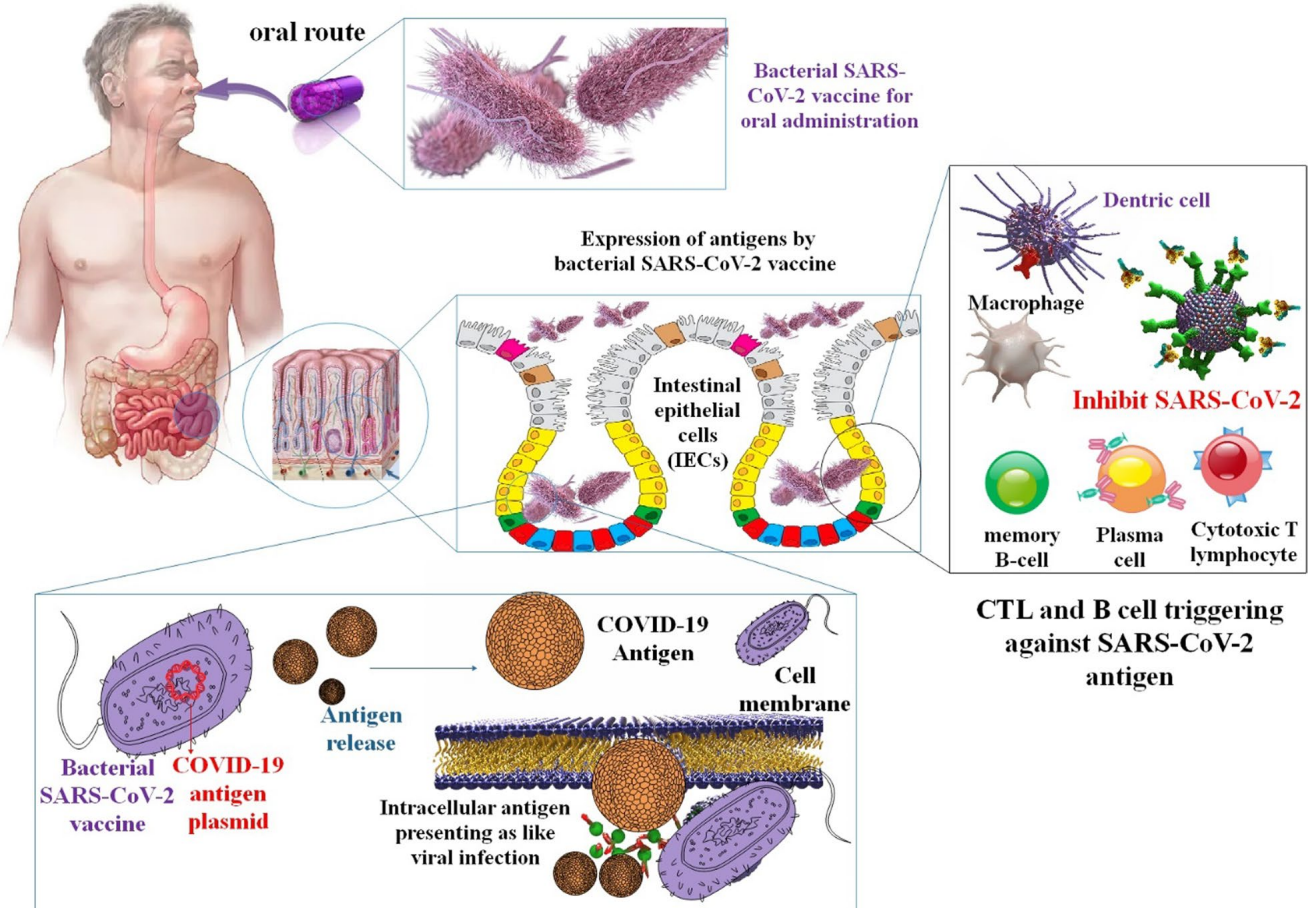
The mechanism by which a bacterial COVID-19 vaccine is administered orally

Vaccines made from *Salmonella* may be given through various methods, including orally and nasally. Even while the gut microbiome may neutralize bacteria-based vaccinations administered orally, the risk of unwanted side effects from the adjuvant or viral vector is thought to be lower with these vaccines. When given orally, *Salmonella* strains are said to exhibit controlled delayed attenuation and stimulate long-lasting humoral and cellular protection. Furthermore, the cost to manufacture vaccines based on attenuated *Salmonella* is minimal. Anti-coronavirus vaccines based on Salmonella are still in their early stages of research; therefore, this study might pave the way for future COVID-19 vaccinations [87]. Another research project looked at whether modified S proteins from SARS-CoV-2 may be used to treat or prevent COVID-19. It has been established that administering these viral proteins using *Salmonella* as a transport platform causes the body to produce antibodies against SARS-CoV-2 and activates cellular immune responses against COVID-19. Furthermore, it was shown that *Salmonella* BRD509, when given orally, did not have significant cytotoxic effects in vivo [88]. In addition, these in vitro and in vivo results support the idea that *Salmonella* strains constitute a reliable platform for the sustained synthesis of antigen proteins because of their efficient type III secretion system. When *Salmonella* infects mucosal cells, it can deliver antigens via intracellular patriotization. More robust cellular and humoral immune responses are produced when the immunization against this virus strain is administered orally. The findings of this study make it possible to create safer *Salmonella* strains that can produce and express antigens of various sizes with fewer inflammatory reactions. These strains could be used as platforms for vaccines with low or no toxicity and the potential for effective antibody production when given orally. Consider Salmonella as a safer oral vaccine development platform for emerging infectious illnesses like COVID-19. Vaccines against COVID-19 could benefit from administration through limoncello strains [83] (Fig. 5).

In an investigation, high-pressure homogenization technology was employed by the researchers to effectively stimulate the bacterial membrane for the production of synthetic vesicles. Additionally, the same technology was utilized to compel the FP cytolysin A (ClyA)-RBD to traverse the apertures in the bacterial membrane, thereby augmenting the interaction between ClyA-RBD and the membrane. By making ClyA look like the support structure of the SARS-CoV-2 S2 protein, researchers could reveal the RBD on the self-assembled bacterial particles very well. High contact, polymerization of ClyA-RBD on bacterial membranes, and the formation of "ring-like" NPs on the vesicles were the results. There was a 107-fold increase in bacterial biomimetic vesicles (BBVs) yield compared to OMV yield, and the RBD was presented on BBVs 28.16-fold more efficiently than on OMVs. Subcutaneously administered RBD-BBVs may localize to lymph nodes, enhance antigen uptake and processing, and stimulate SARS-CoV-2-specific humoral and cellular immune responses in mice [89].

### The utilization of bacteria as a delivery system in HIV

HIV, the virus responsible for acquired immunodeficiency syndrome (AIDS), attacks explicitly CD4+ helper T cells and slowly wipes them out. ssRNA molecules are enclosed by a capsid to form the HIV genome [90]. HIV infections start when the C–C chemokine receptor type 5 (CCR5) or C–X–C chemokine receptor type 4 (CXCR4) coreceptor on target cells come into contact with one of the glycoproteins on the surface of the viral capsid, mainly gp120 [91]. To prevent HIV transmission between heterosexual partners, many methods are being researched. Microbicides delivered topically to the vagina are among those that are being actively investigated. Using a topical microbicide to prevent HIV presents several medication delivery difficulties. The vaginal mucosal barriers and probable medication breakdown in the vaginal lumen owing to pH and enzymes present are some of these difficulties. Additionally, new medications being tested as microbicides have unique modes of action that, in some circumstances, need drug targeting to a particular site of action. In HIV prophylaxis, NPs provide a delivery method for targeted or regulated distribution to the vagina [11].

EcN was engineered to produce HIV-gp41-hemolysin. A synthetic peptide that blocks HIV from entering cells by preventing it from joining with them. Because it may persist in the GI and genital tract of mice for weeks to months, engineered EcN shows promise as an anti-HIV microbicide. In an investigation, Mice afflicted with *V. cholerae* had an improved chance of survival after receiving engineered EcN expressing cholera autoinducer 1. Stable bacterial colonization and intravaginal synthesis of the HIV inhibitor cyanovirin-N were seen after intravaginal delivery of a Lactobacillus strain harboring the gene for cyanovirin-N. Using this method, microorganisms promise as low-cost barriers to HIV transmission [92]. Another study looked at the possibility of employing *S. typhi* Ty21a BGs as a novel method of delivering an HIV vaccine. DNA vaccines offer great promise for inducing immunological responses since they are stable and straightforward to manipulate. Researchers discovered that murine macrophage RAW264.7 cells effectively produced gp140 after exposure to Ty21a BGs loaded with an HIV gp140 DNA vaccine (Ty21a BG-DNA). Anti-gp120 antibody responses were significantly higher in mice immunized with BGs-DNA than in those immunized with DNA alone. BG-induced IL-10 production was linked to Ab responses via the TLR4 pathway. These results suggest that Ty21a BGs is a unique and efficient DNA vaccine delivery vehicle, offering a new approach to creating HIV vaccines. Despite over three decades of research and development efforts, there is presently no HIV vaccine that can be administered to humans. The Ty21a BGs not only stimulated intestinal mucosal immune responses but also enhanced peripheral antibody responses to the pSV140 DNA vaccination. Biased induction of Th2-type cytokines and TLR4 pathway activation seems to be linked to Ty21a BGs’ immune-regulatory effects. Found that HIV-specific IgG and IgA antibodies could be generated in the blood and intestinal mucosa, respectively, using this novel experimental setting [93]. As an additional vector for inducing mucosal immune responses, bacterium-like particles (BLPs) have previously been utilized for several vaccinations against respiratory tract viruses. Mice and guinea pigs were immunized with the vaccine by injecting it into their muscles or giving it to them via their noses. Inducing Env-specific secretory IgA (sIgA) in mucosal locations in mice was shown by administering gp120 trimers bound to BLPs by i.m. injection or i.n. drip. As a result, HIV-1 vaccination using gp120 trimers attached to BLPs shows promise [94].

### The utilization of bacteria as a delivery system in HPV

**Fig. 6:**
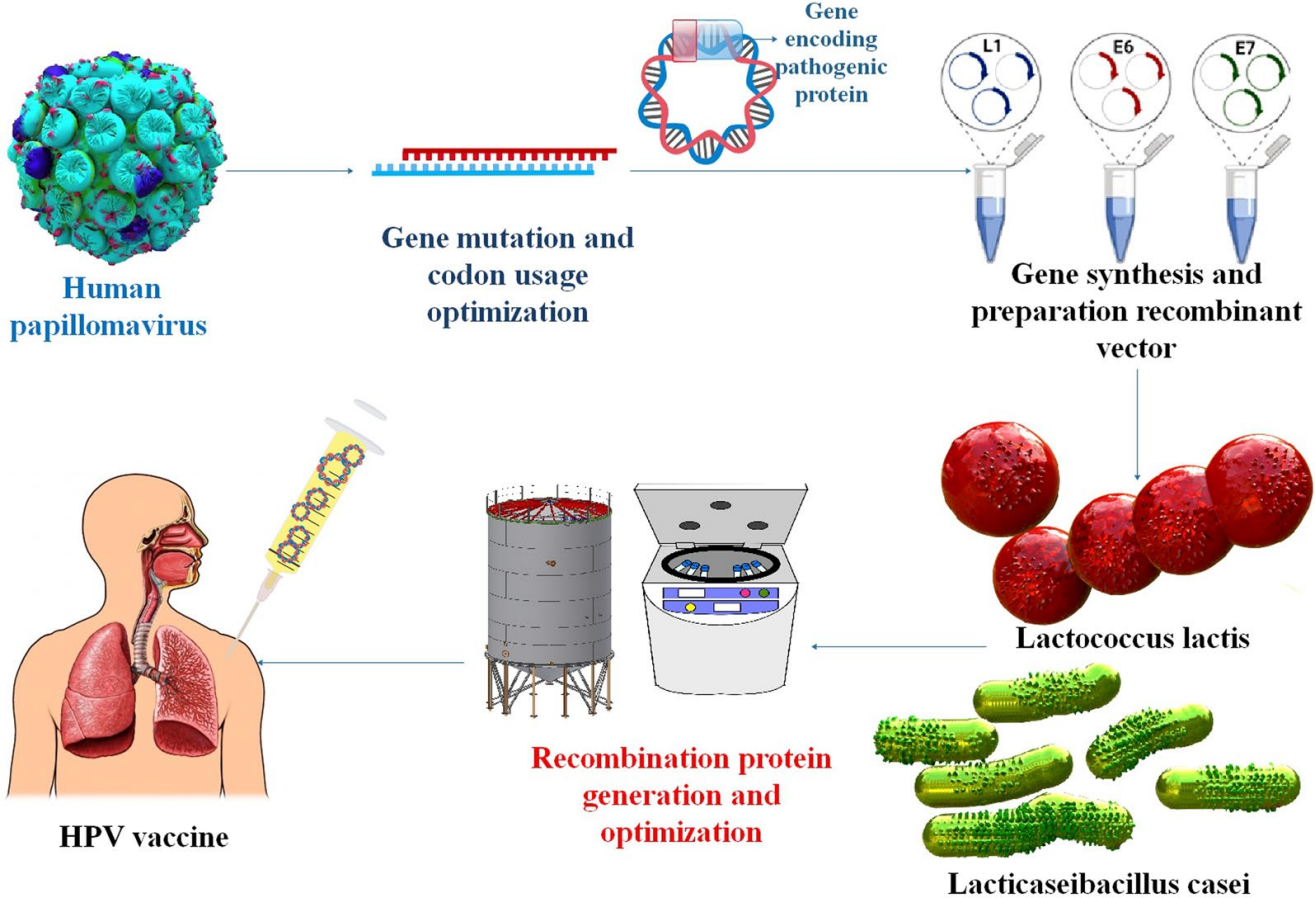
LAB is a typical approach for developing HPV vaccines, and pre-clinical and clinical trial studies have been utilized to determine vaccination effectiveness and safety in mouse models and human test participants

Those who have not yet contracted HPV may benefit from a prophylactic vaccination. That's why it's so important to find ways to treat cancers caused by HPV. Vaccines of the third generation, based on nucleic acids, are a quick and easy way to stimulate adaptive immune responses. For the administration of nucleic acid vaccines, bacteria show great promise as live vectors. LPSs, peptidoglycan, and flagellin are all naturally occurring components of the bacterial vector that are recognized by the immune system and cause it to react strongly [95]. Genetically engineered *lactic acid bacteria* (*LAB*) have been shown in several pre-clinical and clinical experiments to successfully produce mucosal, humoral, and cellular immunity in the host (Table 2) [96]. Taken as a whole, the results of these investigations point to a handful of important considerations that might provide direction toward maximizing protein output and enhancing immunological response. The adaptive immune response is improved, and the native immune response is triggered by this introduction. The particular methods by which bacterial vectors make nucleic acids accessible in host cells are not yet entirely known, however, especially for certain species. Some non-pathogenic bacteria, including *LAB*, and recombinant and attenuated forms of pathogenic bacteria, such as *Salmonella*, *Mycobacterium*, *Yersinia*, *Listeria*, and *Shigella*, are thought to be carriers of nucleic acid vaccines. Although there are benefits to using a bacterial delivery method, such as the production of a robust immune response, oral administration, and improved APC targeting, there are also substantial drawbacks that should be taken into account [47]. So far, many mucosal vaccines targeting HPV-16 L1, L2, E2, E6, and E7 antigens have been produced, all based on genetically engineered LAB. Importantly, studies testing the efficacy of these recombinant LAB in eliciting an immune response have reached both phase I and II of clinical trials [96]. Significant results from another research show that *Bifidobacterium bifidum*, whether given intravenously or orally, successfully promotes antitumor immune responses and reduces tumor development in mice. Intravenous delivery of the probiotic *Bifidobacterium bifidum* to tumor-bearing mice activates tumor-specific IL-12 and IFN-γ, lymphocyte proliferation, and CD8+ cytolytic responses that suppress and eliminate tumor development, in contrast to oral administration alone. These results indicated that immune system modification by probiotics administered intravenously is an efficient anticancer strategy. Additional research on the immunomodulatory effects of the probiotic *Bifidobacterium bifidum* in treating cervical cancer is warranted. The current study set out to compare the preventative-therapeutic impact of oral versus intravenous administration of the probiotic *Bifidobacterium bifidum* on the immune response and tumor growth of C57BL/6 mice harboring transplanted TC-1 cells of HPV-related tumor, expressing HPV-16 E6/E7 oncogenes [97]. Preclinical and clinical investigations of the LAB-based HPV vaccine have led researchers to conclude that LAB is the most promising vehicle for achieving mucosal vaccination goals. The most popular methods for mucosal administration of antigens are the i.n., intravaginal, and oral routes, with the oral route being the most exciting approach for stimulating mucosal immunity by mucosal vaccination. Studies have shown that the most efficient way to induce a mucosal, humoral, and cellular immune response is by oral administration of HPV-16 oncoproteins produced by recombinant *L. lactis* or *L. casei* to the gut mucosa [98] (Table 1) (Fig. 6).

**Table 1:** HPV infection vaccines pre-clinical and clinical trials using *L. lactis* or* L. casei*

Vaccine	Bacteria	Vector	Study type	Administration route and dose	Type of immune response	Refs.
NZ8123-HPV16-optiE7 vaccine	*L. lactis*	HPV-16 E7 oncogene	Phase I clinical trial with 55 individuals, IRCT20190504043464N1	Oral, 20 dose, 1 × 10^9^, 5 × 10^9^, and 1 × 1010 CFU/mL	Antibody levels dropped till day 240, according to the 6 months follow-up, although long-lasting E7-specific IFN-γ-secreting CD8 + CTL responses were seen	[99]
pNZ8123-HPV16-optiE7 vaccine	*L. lactis* NZ9000	pNZ8123	In vitro and in vivo, C57BL/6 mice	Oral, 1 × 10^8^, 10^9^, and 10^10^ CFU	The administration of recombinant *L. lactis* NZ9000 expressing the HPV type 16 E7 antigen via oral route in mice has been shown to elicit significant humoral and cellular immune responses	[100]
BLS-M07	*Lactobacillus casei*	HPV-16 E7 oncogene	A phase 1/2a Clinical Trial with 90 individuals, NCT02195089	Oral, 5 times a week, on weeks 1, 2, 4, and 8, with dosages of 500 mg, 1000 mg, and 1500 mg	The administration of BLS-M07 through oral means results in an elevation of serum HPV16 E7 specific Abs, stimulating the development of humoral immunity that protects against the virus	[101]
HPV16 E7-expressing *L. casei* (GLBL101c)	*Lactobacillus casei*	pIGM2	In vitro and in vivo, C57BL/6 mice	Oral, E7-doses but saturated beyond 0.3 μg/108 cells	The administration of IGMKK16E7 resulted in a four-fold increase in the stimulation of E7-specific mucosal IFNγ-producing cells in comparison to the previous method of immunization	[102]
HPV-16/E6	*L. lactis* NZ9000	pNZ8123	In vitro and in vivo, Female mice of strain C57BL/6	Oral, 1 × 10^9^ CFU	The study involved the oral immunization of female C57BL/6 mice using recombinant *lactococci* expressing inducible E6 oncoprotein. The resulting immune response was evaluated regarding antigen-specific antibody production, including IgA and IgG, as well as humoral, cell-mediated, and mucosal immune responses	[103]
HPV-16/E7 (*L. casei*-E7)	*Lactobacillus casei*	HPV-16 E7 oncogen	In vivo, female C57BL/6 mice, experiments were performed according to the approved guidelines (KRIBB-AEC-16154, KRIBB-AEC-17036)	The administration of 800 μg of γ-PGA (2000 kDa) or PBS via the intragastric route was conducted orally five times per week during weeks 0, 3, 5, 6, and 7. The mice were orally administered with *L. casei* (5 × 109 cells/mouse) or *L. casei*-E7 (5 × 109 cells/mouse) five times per week during weeks 1, 2, 4, and 8	According to the findings, the oral delivery of γ-PGA with *L. casei*-E7 elicits a cooperative antineoplastic outcome, along with humoral, cellular, and mucosal immune reactions	[104]
HPV16 E7-expressing *Lactobacillus* (IGMKK16E7)	*Lactobacillus casei*	HPV-16 E7 oncogene	The present study is a phase I/II randomized trial that is placebo-controlled and double-blind, conducted across multiple centers. Its objective is to evaluate the safety and efficacy of IGMKK16E7 in individuals with HPV16-positive HSIL. The trial involves 41 participants and is registered under the UMIN000034253 and jRCT2031190034	Oral administration, 0.5, 1, and 1.5 g/day	The administration of IGMKK16E7 through the oral route is expected to induce a TH1 mucosal immune response specific to E7	[105]
HPV-16/E7	*L. lactis*	pNZ8123	In vitro and in vivo, C57BL/6 mice	Oral administration, 1 × 10^9^ CFU*/*ml	The administration of recombinant *L. lactis* via oral immunization elicited notable humoral and cell-mediated immune responses in C57BL/6 mice. Specifically, significant increases in specific IgG and IgA Abs were observed in the serum and vaginal fluids, respectively, indicating a robust mucosal immune response	[106]
NZ8123-HPV16-optiE6 vaccine	*L. lactis*	HPV-16 E6 oncogene	Phase I clinical trial with 46 individuals, 20190504043464N1	Oral administration, 1 × 10^9^, 5 × 10^9^, and 1 × 10^10^ CFU/mL	The study observed that the maximum activation of E6-specific IFN-γ-secreting CD8 + CTL responses following oral immunization occurred one month after the final vaccination. The humoral, cellular, and mucosal immune responses are critical components of the immune system	[107]

### The utilization of bacteria as a delivery system in hepatitis viruses

Viral hepatitis continues to pose a significant threat to human health, as it remains a leading cause of mortality on a global scale [108, 109]. Vaccination is the most reliable method of protection against infectious illnesses, and in some instances, it is the only option. Eliminating deadly diseases like smallpox and polio attests to the efficacy of vaccinations in the past. Human liver-infecting viruses are called hepatitis viruses, and there are five main varieties, designated by the letters A through E [110]. Even though hepatitis A virus (HAV) infection is known to self-resolve with rest and symptomatic therapy, there were 7134 HAV-related fatalities globally in 2016. HBV and the hepatitis C virus (HCV) are expected to have caused 820,000 and 290,000 deaths in 2019, respectively. A satellite virus called hepatitis delta virus (HDV) relies on HBV to produce infectious particles to propagate. The most severe chronic viral hepatitis is thought to result from the co-infection of HDV and HBV. Another oral virus that is often seen in low- and middle-income nations is HEV. It contributed to 44,000 fatalities globally in 2015. Hepatitis A and B vaccinations are currently accessible and safe [111].

Around 71 million individuals worldwide are thought to have chronic HCV infection. Investigations demonstrated that there is currently no licensed vaccination against HCV. A specifically synthesized recombinant FP with a shortened core and HCV NS3 (rC/N) has been expected to be stable and immunogenic. Recombinant proteins are harmless antigens, but without adjuvants, they do not make effective vaccinations [113]. In the recent investigation, BALB/c mice were used to assess the immunogenicity of rC/N as a bipartite antigen combined with *N. meningitidis* serogroup B OMVs (NMB OMVs). Compared to MF59 and Freund’s CIA, NMB OMVs significantly improved Th1 immune responses. A potential method for creating a therapeutic HCV vaccination is rC/N-NMB OMVs. Recombinant protein vaccines are safe and effective, although they often only provide mediocre immunity. Therefore, one or more immune-stimulatory components must be included in the vaccine formulation to stimulate immunological responses that are strong, focused, and persistent in response to the delivered antigen. Microbially derived adjuvants stand out among those legally allowed to be used. The OMVs, which are primarily generated from Gram-negative bacteria, are inherently non-replicating and highly immunogenic. Vesicles produced by detergents may produce various pro-inflammatory cytokines and are safe to use. *N. meningitidis* serogroup B OMVs (NMB OMVs) have shown that they may be used as adjuvants in various bacterial and viral vaccines to enhance cellular and humoral immune responses. For cancer immunotherapy in mice, the OMVs of *E. coli* are also employed as an adjuvant and delivery mechanism. To assess the likely immune responses to HCV in a mouse model, this work administered the NMB OMVs simultaneously with the shortened recombinant core1-118 (rCore) and NS31095-1384 (rNS3) FP (rC/N) [114].

HBV virus is an enveloped virus with DNA-based genetic information encapsulated within an icosahedral capsid [112]. The HBcAg-149 was used as a model antigen in a different study to assess and compare the immunogenic capacity of recombinant ghosts employing inner and OM anchor systems. Delivery system particle production may facilitate antigen absorption by APCs, particularly dendritic cells, at the site of infection. These cells subsequently move to lymph nodes where the antigen is presented to naïve T-cells. Empty bacterial cell ENVs (ghosts) were studied for their potential to transport exogenous antigens via two membrane compartments of *E. coli*. Mice were immunized subcutaneously with BGs containing the HBV core 149 protein (HBcAg-149) as a model antigen, and their immune responses were compared to those of BGs containing a control antigen anchored in the inner membrane of *E. coli*. Mice exposed to either system developed robust immune responses against the HBcAg-149 target antigen. The findings demonstrate that BGs function splendidly as a vehicle for transporting antigens. By inserting an E-specific transmembrane tunnel structure into their cell wall, Gram-negative bacteria produce BGs when the cloned lysis gene E of bacteriophage PhiX174 is expressed [37].

Several attenuated strains of *S. Typhimurium* and *S. Dublin* were found to stably produce hybrid HBV nucleocapsid-pre-S(2) FPs in the presence of aromatic compounds. When these live recombinant bacteria were injected intraperitoneally (i.p.) into BALB/c mice, they produced antibodies against the HBV core antigen (HBc) and the pre-S2 protein. Following oral administration of recombinant salmonellae, the seroconversion rate to anti-HBc in mice differed by salmonella strain. High titer anti-HBc Abs and lower titer anti-pre-S2 serum were seen in the best carrier strain two weeks after a single oral vaccination. The distribution of Ig class and IgG subclass anti-HBc Abs after intramuscular and oral vaccination is supportive of the induction of functional T cell assistance [115].

### The utilization of bacteria as a delivery system in influenza virus

A case of the flu, despite seasonal flu vaccination, a virus experiences genetic drift and change, making the general population vulnerable to newly developing pandemic strains. Recombinant OMVs (rOMVs), generated from E. coli, have been suggested as a potentially helpful vaccination strategy that directly connects adjuvant and antigen. OMVs (diameter: 50–200 nm) displaying the target antigen are shed when hyper vesiculating strains of E. coli are transformed with a plasmid containing the transmembrane protein ClyA followed by the target antigen [116]. The rOMVs can be harvested, suspended in a buffer, and employed as a vaccine, obviating additional protein purification or the inclusion of supplementary adjuvants, which is a prerequisite for other expeditious vaccine platforms. The research team has demonstrated that rOMVs containing a heterospecies tandem sequence of peptides derived from the matrix 2 protein ectodomain of influenza (M2e4xHet), comprising of human, swine, and avian species, have the potential to safeguard against various influenza A subtypes in mice with distinct genetic backgrounds. This finding establishes M2e4xHet rOMVs as a promising vaccine candidate for protecting against pandemic influenza A [117]. The study conducted by the researchers demonstrated that the regulated discharge of rOMV constructs exhibits promise as a unipotent vaccine to safeguard against the influenza A challenge. The vaccine generates antibody titers expeditiously, which endure for at least 6 months in mice, thereby providing protection. The utilization of PLGA µP containing M2e4xHet rOMVs has been observed to yield substantial and enduring immunity against the influenza A/PR8 challenge. Prior research involving PLGA µP has involved encapsulating inactivated influenza virus, influenza antigens, and influenza DNA for vaccine development against influenza [116]. The activation of TLR signaling through transported pathogen-associated molecular patterns (PAMPs) occurs upon the uptake of OMV by host cells. Alveolar macrophages are a crucial type of immune cells situated at the interface between air and tissue. They play a pivotal role in defending against inhaled microorganisms and particles, serving as the primary line of defense. The present investigation examined the reaction of primary human macrophages to bacterial vesicles, namely *Legionella pneumophila*, *Klebsiella pneumoniae*, *E. coli*, *S. enterica*, and *Streptococcus pneumoniae*. The researchers observed that there was a similar level of NF-κB activation across all the vesicles that were tested. In contrast, scholars depict a distinctive IFN-I signaling pattern characterized by extended STAT1 phosphorylation and robust Mx1 induction, which impedes the replication of influenza A virus solely for *Klebsiella*, *E. coli*, and *Salmonella* OMVs. The antiviral effects induced by OMVs were observed to be comparatively weaker for Clear coli OMVs that were devoid of endotoxin and OMVs that were treated with Polymyxin. The antiviral status was not replicated by LPS stimulation; however, it was nullified by the absence of TRIF due to knockout. Significantly, the supernatant obtained from macrophages treated with OMV elicited an antiviral reaction in alveolar epithelial cells (AEC), indicating the occurrence of intercellular communication induced by OMVs. Ultimately, the findings were verified through an ex vivo infection model utilizing primary human lung tissue. To summarize, the OMVs of *Klebsiella*, *E. coli*, and *Salmonella* prompt antiviral immunity in macrophages through TLR4-TRIF signaling, thereby diminishing viral replication in macrophages, AECs, and lung tissue. Gram-negative bacteria have been observed to stimulate antiviral immunity in the lungs via OMVs. This finding has significant implications for the outcome of bacterial and viral coinfections [118].

Others have also proven that *LAB*, depending on the strain, may alter respiratory immunity when taken orally. Using a fatal model of influenza virus pneumonia, the ability of many *L. plantarum* strains to modify respiratory immunity when orally delivered was assessed. The *L. plantarum 06CC2* strain was the most effective in increasing the survival rate of influenza-infected mice, whereas the *L. plantarum 05AM23*, *06TCa8*,* 06TCa4*0, and *06CC9* strains had no impact. The administration of the 06CC2 strain through oral means resulted in a decrease in influenza virus titers in the lungs. Additionally, it was observed that there was an improvement in the Th1 response in the respiratory tract, as well as an increase in NK-cell activity in both the lungs and spleens. Furthermore, a comprehensive screening of immunomodulatory *LAB* was conducted, wherein multiple strains of *Lactiplantibacillus* were assessed for their capacity to modulate immunity in TNF-α-activated HT-29 cells and peripheral blood mononuclear cells. Of the strains that were evaluated, *L. plantarum CNRZ1997* exhibited a noteworthy ability to augment the production of cytokines associated with inflammation in both epithelial and immune cells. Conversely, other strains of *L. plantarum* either had no impact or displayed anti-inflammatory properties. The study revealed that the CNRZ1997 strain, when administered orally, could diminish the proliferation of the influenza virus in the respiratory tract of mice. The efficacy of oral administration of *L. plantarum CNRZ1997* was comparable to that of *L. rhamnosus GG* or *L. casei DN114-001*, two commercially available probiotic strains that have demonstrated anti-influenza virus properties in preclinical and human trials, in safeguarding mice against influenza infection [119–122].

### The utilization of bacteria as a delivery system in another virus

Ebola virus (EV), belonging to the Filoviridae family, is a zoonotic pathogen responsible for causing Ebola virus disease (EVD), also called Ebola hemorrhagic fever. The disease is endemic to West Africa and Middle Africa and has resulted in over 34,000 human cases and 15,000 fatalities. Viruses from Zaire (EBOV), Bundibugyo (BDBV), Sudan (SUDV), Tai Forest (TAFV), Reston (RESTV), and Bombali (BOMV) are only some of the six antigenically different species that make up the EV genus. A substantial health danger is posed by EBOV, SUDV, and BDBV because of the high mortality rates associated with their associated diseases in humans (up to 90% in some instances) [123]. In West Africa and Middle Africa, SUDV and EBOV cause serious sickness and, on occasion, death. The BLPs vaccines can potentially provide immunized people with safer and more effective protection against infections. Subunit vaccines have extensively used BLPs, a unique surface display technology for proteins. Nonliving particles generated from gram-positive *L. lactis* used in food production that have not been genetically changed are the basis of the BLPs surface display technology, which also uses a protein anchor (PA). *L. lactis* is well suited for use as a vaccine since it has a history of being considered safe by the FDA. AcmA is an autolysin from *L. lactis*, and its C-terminal peptidoglycan-binding domain provides three lysin motifs (LysM) for the PA [124]. This research demonstrates the immunogenicity in mice of a bivalent BLPs-based vaccination created by combining SUDV-BLPs with EBOV-BLPs at a 1: 1 ratio, called SUDV-EBOV BLPs (S/ZBLP + 2 + P). The SUDV-EBOV BLPs generated both Th1 and Th2 immune responses and caused strong protection against SUDV and EBOV. The findings suggested that a vaccine based on SUDV and EBOV BLPs might be a strong contender in the fight against SUDV and EBOV infections and provide a method for creating universal vaccinations against EVD [123].

The predominant etiological agent of non-bacterial gastroenteritis on a global scale is the human norovirus. In developing nations, it is the second most common cause of diarrheal fatalities in children. Empirical data indicates that noroviruses can adhere to the exterior of commensal bacteria, and the existence of these bacteria has an impact on both acute and persistent murine norovirus infection by influencing the host's immune responses. The interactions between norovirus and bacteria have been found to induce stress responses in the bacteria and result in an increase in the production of BEVs. The researchers postulated that BEVs may impact murine norovirus infection by modulating the antiviral immune response based on their known capacity to readily traverse intestinal barriers and penetrate the lamina propria to modulate host responses. The current investigation demonstrates the capacity of murine norovirus to adhere to purified bacterial vesicles, thereby enabling the simultaneous introduction of virus and vesicle to target cells. Investigations employed *S. Typhimurium* to investigate the impact of OMVs originating from an intestinal pathogen in contrast to those generated by commensal bacteria. In addition, scholarly investigations have demonstrated that the co-inoculation of macrophages with murine noroviruses and vesicles results in a decrease in viral infection as compared to the infection caused by the virus alone. Co-inoculation with bacterial vesicles leads to increased production and release of pro-inflammatory cytokines in response to viral infection. The findings suggest that bacterial vesicles could potentially function as a means of regulating and curtailing murine norovirus infection, as evidenced by the increased bacterial vesicle production in vivo following infection. This may ultimately serve to restrict the duration of the disease [125] (Table 2).

**Table 2:** Bacteria and bacterial derivatives as Drug delivery systems in viral infection

Viral infection	Delivery system	Use	Effects	Refs.
SARS-CoV-2	*E. coli*	SARS-CoV-2 FP vaccine	The FP can potentially serve as a viable target for a coronavirus vaccine with broad protective capabilities. This assertion is based on the observation that a vaccine targeting the betacoronavirus SARS-CoV-2 FP was able to confer cross-protection against the alphacoronavirus PEDV	[28]
SARS-CoV-2	OMV derived from *S. Typhimurium*	RBD-OMV vaccine	High levels of anti-RBD IgG were found in the blood after intranasal immunization, and there were also clear mucosal reactions. Upon challenge with live virus, hamsters immunized with RBD-OMV avoided body mass loss, had lower viral titers in bronchoalveolar lavage fluid, and exhibited less severe lung pathology than those immunized with unconjugated OMVs or vehicle control	[85]
SARS-CoV-2	attenuated *Salmonella* strain	oral vaccines for delivery of sipB160 protein	This strain was created to produce SARS-CoV-2 antigens using the sipB160 protein for synthesis or partial secretion. Intestinal macrophages with high bacterial cell viability may be activated into B- and T-cells by ingesting this *Salmonella*, making it useful as a vaccine. This vaccine’s oral administration and use of this viral strain have the potential to elicit more robust cellular and humoral immune responses	[83]
SARS-CoV-2	OMVs from *N. meningitidis*	OMV-mC-Spike vaccine	Vaccination with OMV-mC-Spike elicited serum-neutralizing Abs in all animals. Immunization through the intranasal route only elicited an IgG response in the blood, whereas intramuscular immunization caused an IgG response in the nose and lungs	[84]
HIV	*Lactobacillus*	Intravaginal administration of HIV inhibitor cyanovirin-N	When a *Lactobacillus* strain expressing the HIV inhibitor cyanovirin-N was inserted vaginally, it established a stable bacterial colony and began producing cyanovirin-N there	[92]
HIV	*Escherichia coli Nissle 1917*	Delivery of HIV-gp41-hemolysin A hybrid peptides	There is hope that engineered EcN might serve as an effective anti-HIV microbicide since it can colonize the gastrointestinal tracts and vaginas of mice for weeks to months. Mice afflicted with *V. cholerae* had an improved chance of survival after receiving engineered EcN expressing cholera autoinducer 1	[92]
HIV	*S. typhi* Ty21a BGs	HIV gp140 DNA vaccine	The murine macrophage RAW264.7 cells exhibited high uptake efficiency of Ty21a BG-DNA, leading to proficient expression of gp140 within the cells. The BGs-DNA vaccine elicited notably elevated anti-gp120 Ab responses in the peripheral and intestinal mucosal regions of mice in comparison to the naked DNA vaccine	[93]
HBV	*E. coli* as a BGs	HBcAg-149 as a model antigen	The study's findings on the immune responses directed towards the foreign target antigen, HBcAg-149, in mice suggest that BGs serve as a highly practical carrier system for the delivery of antigens	[37]
HBV	*S. Typhimurium* and *Salmonella Dublin*	Hybrid HBV nucleocapsid-pre-S(2) fusion proteins	Upon intraperitoneal administration to BALB/c mice, these live recombinant bacteria elicited a significant increase in titer of anti-HBV core antigen (HBc) and detectable levels of anti-pre-S2 serum. Abs	[115]
Influenza	*E. coli*	rOMVs as a vaccine	The potential of rOMV constructs for controlled release makes them a promising candidate for a single-dose influenza vaccine. The study aimed to investigate the feasibility of achieving a rapid generation of antibody titers that exhibit protective efficacy for a minimum duration of six months in murine models	[117]
Influenza A	*E. coli*	M2e4xHet rOMVs vaccine	Using PLGA µP containing M2e4xHet rOMVs demonstrated notable and enduring safeguarding against the influenza A/PR8 challenge. Prior research involving PLGA µP has involved encapsulating inactivated influenza virus, influenza antigens, and influenza DNA for vaccine development against influenza	[116]
Influenza	*L. plantarum*	Orally administered to modulate the respiratory immunity	The strain *L. plantarum 06CC2* was observed to decrease influenza virus titers in the lungs and enhance the Th1 response in the respiratory tract, as well as augment NK-cell activity in both the lungs and spleens	[119–122]
EBOV	*L. lactis*	BLPs vaccines	The findings suggest that the vaccine based on SUDV-EBOV BLPs exhibits promise as a potential contender against infections caused by SUDV and EBOV. Additionally, it presents a viable approach for developing universal vaccines for EDV	[123]

## Advantages and disadvantages of bacterial drug delivery system in viral infection

Microbial-mediated drug delivery has emerged as a promising therapeutic approach for various ailments, including viral infections. A novel strategy involving the utilization of microbes for drug delivery with reduced toxicity and side effects holds promise as an advanced carrier for enhancing patient health in the future [126]. The absence of precise mechanical knowledge of the connections between illnesses and bacteria is a constraint on developing live-engineered microbes as therapeutic agents. Blood-borne bacterial therapies call for elucidation of the correlation between the pharmacodynamics of the target illness and the ideal quantity of medication, which is the total of the proliferating bacterial density and the concentration of delivered cargo medicine. Even while this could improve methods using modified therapeutic bacteria, little is now understood about the pharmacokinetics and dose-response interactions of these bacterial agents in the human body. The last factor that may improve the compatibility of modified strains with their human hosts is the development of new synthetic biology methods that allow commensal bacteria to be considered carriers for created bacterial medicines. *E. coli*, *Salmonella*, and *Lactobacillus* strains benefit most from the current state of synthetic biology tools for bacteria, whereas obligate anaerobes and other Gram-positive bacteria have far less access [61]. Moreover, although bacterial delivery systems offer various benefits, including the potent induction of immune response, oral administration, and enhanced targeting of antigen-presenting cells, they also entail notable drawbacks that necessitate careful consideration. Furthermore, bacterial delivery mechanisms, such as viruses, generate neutralizing Abs against the bacterial vector, leading to a reduction in the effectiveness of the vaccine antigen [127].

*L. lactis*, *S. typhi*, and *E. coli Nissle* are the modified microbes studied and used as medication carriers the most extensively. Cancer, autoimmune illnesses, metabolic problems, and inflammatory conditions are only some of the diseases for which these have been used in vaccines. *Bifidobacterium animalis*, *L. monocytogenes*, *Staphylococcus epidermidis*, *Staphylococcus lugdunensis*, and *Clostridium sporogenes* are among more strains that show promise. The limited effectiveness and rapid clearance caused by immunological responses generated by high bacterial concentrations are key downsides, despite the minimal risk observed. Thus, a better and more effective mode of medication administration is required, and additional study in this field is also required combining bacterial-based medicines with other current treatments [128].

Since modified bacteria are alive and can carry out a wide range of biological functions once inside the body, they offer significant advantages over more conventional medication delivery methods. Current medication delivery technologies cannot duplicate this kind of exact response, which requires interaction with the microenvironment to achieve. Biological processes can be naturally occurring or artificially introduced into the preferred bacterial strain. In addition to functioning as microbial cell factories, engineered probiotics have been augmented with sensors and response systems through genetic engineering. This allows live bacteria to effectively transport therapeutic proteins or molecules to various regions of the human body, including the gastrointestinal tract, oral and intranasal cavities, and the skin. The human body provides a natural home for various microorganisms, with bacteria constituting the majority of the microbiota, making these treatments more convenient to give. These bacteria establish permanent colonies in specific anatomical sites with optimal circumstances for their survival. Symbiotic and pathogenic bacteria both use biochemical signaling to communicate with their hosts. To combat illness, scientists have modified commensal and weakened harmful bacteria to transport therapeutic chemicals [129].

BEVs have been used as a novel method of administering medication. Further stumbling blocks to BEV research development include a lack of standardized procedures for separation and purification, as well as a lack of well-defined identification of particular markers present on BEVs. There is a lack of data on maximizing BEV production from fermentation broth with its complicated medium composition and metabolites. Overexpression or deletion of specific genes may affect EV secretion and design, and gene editing technology has seen extensive usage in the bacterial realm. We hope that by writing this evaluation, you will see that BEVs have potential beyond only treating viral infections when it comes to medication delivery. However, the clinical translation of BEVs as delivery systems or treatments still faces considerable obstacles, such as possible biosafety issues, expensive and time-consuming isolation procedures, and content and mechanism ambiguity [44]. Genetic engineering, cargo loading, and NP coating are just a few ways that BMVs might be transformed into multifunctional platforms for use against a wide range of illnesses [40].

As a novel drug delivery technology, BGs exhibit a wide range of biological properties. BGs are amenable to industrial-scale manufacture because of how readily they accept nucleic acids, proteins, and chemicals. Because BGs are devoid of DNA, the possibility of horizontal gene transfer is eliminated. In addition to improving the immunogenicity and treatment efficacies of medications, BGs’ status as bacterial cell wall enclosures means that they may express and be loaded with various antigens or pharmaceuticals. BGs may exclude certain cell types, such as APCs, HCDECs, and Caco-2. Tumor immunotherapy and vaccines may benefit from BGs because of their immunogenicity and targeting. Many medications lose some of their effectiveness when taken orally. The use of BG-based delivery methods may be able to circumvent this problem. However, BGs’ therapeutic services come with their own unique set of difficulties. For instance, further research has to be done into the therapeutic benefits of combining BGs of various strains with antigens or medicines. Additionally, it is essential to plug any leaky holes in BGs and study the stability and processes of combining different medications or antigens with BGs. Since the immune system readily eliminates BGs in vivo, it is crucial that their targeting and stability be enhanced [60].

*Salmonella* strains, for instance, have shown promise as a potential novel delivery method for COVID-19 vaccines, according to in vitro and in vivo studies. These adverse effects may have resulted from excessive dosing with lipid NPs (the vehicle of delivery) used to introduce antigen genes into muscle cells, or with immunostimulant (adjuvant) chemicals intended to boost particular immunity. Therefore, while being a bacterium (*salmonella*), it has the features of generating mucosal immunity and the expression of antigens (for example, MHC class I) in cells because it possesses an infection mechanism comparable to that of a virus. Attenuated Salmonella-based vaccines also have a cheap cost of manufacture. Antigen-specific T-cell activity and CTL responses, as well as antiviral immune responses (such as interferon-γ), may be induced by this altered Salmonella-based vaccination method without the addition of any additional immunoactivity agents, which is a significant plus. In sum, *Salmonella* has the potential to serve as a more secure oral vaccine development platform for emerging infectious illnesses such as COVID-19 [83]. The administration of *Salmonella* and *Listeria* vaccines has been associated with various unfavorable outcomes. Conversely, the utilization of *LAB* for HPV vaccination has been correlated with a lower incidence of adverse effects. This suggests that *LAB* vaccination may serve as a viable and promising substitute approach to conventional attenuated pathogenic bacterial immunization [130]. Although there are currently no reported BMV-based vaccines for preventing HPV, there exists a possibility for future development in this area. Despite extensive endeavors by various groups in the last two decades, there is a lack of knowledge regarding how the regulation of gut microbiota through mucosal *LAB*-based vaccine and its resultant signals to the vagina safeguard the female genital tract against HPV. Despite our knowledge of the modulatory impact of *LAB*-derived vaccine on HPV in CxCa, several aspects require further elucidation. It is noteworthy that certain studies suggest that the development of active immune deviation to E6/E7 CMI responses has likely occurred in most patients with advanced HPV-induced lesions [96]. Subsequently, the antigen was combined with purified *N. meningitidis* BMVs and subcutaneously injected into mice. formulation as mentioned above, demonstrated the ability to elicit elevated levels of IgG antibodies, proinflammatory cytokines, and granzyme B, thereby suggesting its potential as a candidate for an HCV vaccine. A vaccine targeting HBV was created utilizing a BMV approach, whereby the HBV core protein was affixed to either the inner or OM of *E. coli* [40, 131].

The majority of existing literature on the utilization of weakened bacterial vaccine carriers primarily focuses on the creation of systems in which the foreign protein antigen is encoded on a plasmid. Given the increasing apprehension surrounding the release of genetically modified organisms (GMOs) that possess antibiotic resistance markers, regulatory agencies are expected to exert significant pressure to discourage the incorporation of such features in live attenuated vaccine strains. Plasmids should include specific confinement characteristics as an extra safety attribute to reduce transmission to and establishment in other bacterial species. Therefore, the ideal plasmid vector should be nonconjugative, preferably non-mobilizable, have a limited range of replicon hosts, and not carry any markers for antibiotic resistance [132]. Antibiotic resistance genes (ARGs) are often used as selection markers in expression systems. They are one of the leading causes for worry due to the potential for spreading ARGs to microbial communities in the host microbiota or the environment. An alternate approach to addressing these issues is the deployment of food-grade systems, which may be based on auxotrophy, complementing markers, or the creation of biocontainment techniques [133].

## Conclusion

With the development and clinical evaluation of novel therapeutic agents for viral infections, there is an urgent need to identify safe and effective delivery platforms that enable the controlled release of these agents. Bacteria that have been modified through engineering, as well as their derivatives, can detect signals within the host, react at the location of the ailment, and administer viable therapeutics that can alleviate issues linked to systemic exposure and toxicity. Engineered microorganisms have the potential to function as biological thermostats, producing therapeutic effectors solely in response to demand. The utilization of genetically modified organisms in food has raised concerns; however, their application in medicine is expected to be more widely accepted, particularly in treating diseases that are currently incurable. Furthermore, there is a considerable ongoing investigation into using bacteria for drug delivery, which has demonstrated promising outcomes in both preclinical and clinical settings. A novel strategy involving using microbes as drug carriers with reduced toxicity and side effects holds promise as an advanced approach to enhance patient health in the future actively. Additionally, the containment of GMOs is a significant issue in their utilization. Plasmid-free bacterial strains must be employed in clinical studies to avoid horizontal gene transfer. Due to in vivo instability over time, plasmid-encoded systems could potentially exhibit a distinct dynamic behavior. If this instability is not managed, it may result in unwanted dose effects. It is therefore recommended to remove the integrase genes before integrating the synthetic systems into genomic DNA. This review demonstrates that bacteria and their derivatives have the potential to serve as effective carriers for genes, vaccine compounds, and proteins in various viral diseases, including but not limited to SARS-CoV-2, HBV, HCV, HIV, HPV, influenza, and EV. The clinical implementation of bacterial-based delivery systems in the context of viral infections is accompanied by several challenges. However, noteworthy obstacles exist to the clinical performance of bacterial-mediated delivery systems in the context of viral infections. These challenges encompass intricate and time-consuming isolation procedures, intricate and ambiguous contents, potential biosafety and regulatory concerns, dose-dependent toxicity, the possibility of DNA mutations that could undermine the efficacy of the therapy, and the need to elucidate the mechanism underlying the severity of systemic infections. It is imperative to explore diverse bacterial strains as potential delivery mechanisms for distinct viral infections.

## Data Availability

Not applicable.
